# Conformational quiescence of ADAMTS‐13 prevents proteolytic promiscuity

**DOI:** 10.1111/jth.13445

**Published:** 2016-09-23

**Authors:** K. South, M. O. Freitas, D. A Lane

**Affiliations:** ^1^ Centre for HaematologyImperial College LondonLondonUK

**Keywords:** ADAMTS‐13 protein, human, allosteric regulation, fibrinogen, fibrinolysis, protein conformation, von Willebrand factor

## Abstract

**Essentials**

- Recently, ADAMTS‐13 has been shown to undergo substrate induced conformation activation.
- Conformational quiescence of ADAMTS‐13 may serve to prevent off‐target proteolysis in plasma.
- Conformationally active ADAMTS‐13 variants are capable of proteolysing the Aα chain of fibrinogen.
- This should be considered as ADAMTS‐13 variants are developed as potential therapeutic agents.

Click to hear Dr Zheng's presentation on structure function and cofactor-dependent regulation of ADAMTS‐13

**Summary:**

**Background**

Recent work has revealed that ADAMTS‐13 circulates in a ‘closed’ conformation, only fully interacting with von Willebrand factor (VWF) following a conformational change. We hypothesized that this conformational quiescence also maintains the substrate specificity of ADAMTS‐13 and that the ‘open’ conformation of the protease might facilitate proteolytic promiscuity.

**Objectives**

To identify a novel substrate for a constitutively active gain of function (GoF) ADAMTS‐13 variant (R568K/F592Y/R660K/Y661F/Y665F).

**Methods**

Fibrinogen proteolysis was characterized using SDS PAGE and liquid chromatography‐tandem mass spectrometry (LC‐MS/MS). Fibrin formation was monitored by turbidity measurements and fibrin structure visualized by confocal microscopy.

**Results**

ADAMTS‐13 exhibits proteolytic activity against the Aα chain of human fibrinogen, but this is only manifest on its conformational activation. Accordingly, the GoF ADAMTS‐13 variant and truncated variants such as MDTCS exhibit this activity. The cleavage site has been determined by LC‐MS/MS to be Aα chain Lys225‐Met226. Proteolysis of fibrinogen by GoF ADAMTS‐13 impairs fibrin formation in plasma‐based assays, alters clot structure and increases clot permeability. Although GoF ADAMTS‐13 does not appear to proteolyse preformed cross‐linked fibrin, its proteolytic activity against fibrinogen increases the susceptibility of fibrin to tissue‐type plasminogen activator (t‐PA)‐induced lysis by plasmin and increases the fibrin clearance rate more than 8‐fold compared with wild‐type (WT) ADAMTS‐13 (EC50 values of 3.0 ± 1.7 nm and 25.2 ± 9.7 nm, respectively) in *in vitro* thrombosis models.

**Conclusion**

The ‘closed’ conformation of ADAMTS‐13 restricts its specificity and protects against fibrinogenolysis. Induced substrate promiscuity will be important as ADAMTS‐13 variants are developed as potential therapeutic agents against thrombotic thrombocytopenic purpura (TTP) and other cardiovascular diseases.

## Introduction

ADAMTS‐13 is a multi‐domain glycoprotein that proteolyses the A2 domain of von Willebrand factor (VWF) and regulates its hemostatic function 1, 2, 3, 4. Multiple VWF‐binding exosites have been identified across a number of ADAMTS‐13 domains 5, which have informed the development of a so‐called ‘molecular zipper’ model of interaction and proteolysis 6, 7, 8, 9, 10, 11, 12, 13, 14, 15, 16. An interaction occurs between ADAMTS‐13 and globular VWF, in which the distal C‐terminal tail of ADAMTS‐13 and the C‐terminal D4‐CK domains of VWF make contact 17. This moderate affinity binding (K_D_ of ~80–120 nm) interaction has been described as a positioning one, allowing a small proportion of ADAMTS‐13 to circulate in complex with VWF 5, 18. Much tighter binding occurs following A2 domain unfolding, between the ADAMTS‐13 spacer domain exosites (Arg659, Arg660, Tyr661 and Tyr665) and the newly exposed VWF A2 residues Asn1651‐Arg1668 13. Next, exosites in the cysteine‐rich domain (Gly471‐Val474) and the disintegrin‐like domain (Arg349 and Leu350) of ADAMTS‐13 interact with complementary binding sites in the A2 domain, progressively closer to the cleavage site 9, 12. This facilitates positioning of the ADAMTS‐13 metalloprotease domain over the VWF scissile bond, with the ADAMTS‐13 S3 subsite (Leu198, Leu232 and Leu274) binding to the VWF P3 residue Leu1603 10. The ADAMTS‐13 active site contains a 3xHis Zn^2+^ binding motif and catalytic Glu residue that are flanked by S1 and S1’ pockets, which specifically bind the P1 (Tyr1605) and P1’ (Met 1606) residues of the VWF scissile bond, leading to proteolysis 15.

There are three properties of ADAMTS‐13 that are unusual. Firstly, it is secreted and circulates as an active enzyme 19, 20. Secondly, ADAMTS‐13 has a long plasma half‐life (2–3 days) and has no known physiological inhibitors 21. Thirdly, ADAMTS‐13 appears to display no off‐target proteolysis, acting only on VWF. The first of these properties has, until recently, been explained by the dependence of ADAMTS‐13 proteolytic function on VWF conformation and the exposure of its complementary binding sites. However, it has recently been shown that ADAMTS‐13 undergoes its own conformational change in order to attain a fully active state 22, 23. In this new model of ADAMTS‐13 function the enzyme circulates in a ‘closed’ conformation mediated by binding between its spacer and CUB domains 22. Upon binding to the D4‐CK domains of globular VWF, ADAMTS‐13 is induced to adopt an ‘open’ conformation exposing the cryptic spacer domain exosites (this conformational change also exposes the autoantibody epitopes recognized in thrombotic thrombocytopenic purpura) 22. Some ADAMTS‐13 variants, such as the gain of function (GoF) spacer domain variant first described by Jian *et al*. 24, are in a pre‐activated ‘open’ conformation 22.

The finding that ADAMTS‐13 circulates in a ‘closed’ conformation explains how it can be secreted as an active enzyme, with its proteolytic potential only being achieved upon binding to its substrate. This may also explain the substrate specificity of ADAMTS‐13, because in a ‘closed’ conformation the active site of the enzyme may not be accessible to additional substrates. However, when ADAMTS‐13 adopts its ‘open’ conformation it may be able to proteolyse other proteins at the site of vascular injury. In this report, we demonstrate that conformational activation of ADAMTS‐13 reveals its ability to proteolytically cleave fibrinogen.

## Methods

### Fibrinogen proteolysis examined by SDS PAGE and Western blot

ADAMTS‐13 variants were expressed in HEK293S stable cell lines as previously described (15) and purified by immunoaffinity using α‐c‐myc agarose (Thermo Fisher, Waltham, MA, USA). Fibrinogen, purified from human plasma, was purchased from Sigma‐Aldrich (St. Louis, MO, USA). ADAMTS‐13 was pre‐incubated at 37 °C for 1 h in the presence of 5 mm CaCl_2_ prior to the addition of fibrinogen to a final concentration of 1 mg mL^−1^. Reactions were incubated at 37 °C and stopped after 180 min by the addition of SDS PAGE loading buffer. Samples were run on 4–12% BIS‐TRIS gels in MOPS buffer (Invitrogen, Carlsbad, CA, USA) and stained with Coomassie or transferred to nitrocellulose for Western blot using a pAb against the Aα chain (residues 21–320) of human fibrinogen (Abcam, Cambridge, UK).

### Mass spectrometry

For MALDI‐TOF mass spectrometry, fibrinogen (1 mg mL^−1^) was digested in solution with 50 nm GoF ADAMTS‐13 overnight at 37 °C. Reduced and non‐reduced samples were applied to C18 tips (Thermo Fisher) and eluted in 0.1% Trifluoroacetic acid in 95% acetonitrile. Using sinapinic acid as matrix, samples were analyzed using an Applied Biosystems (Foster City, CA, USA) Voyager DE Pro Biospectrometry workstation and DataExplorer processing software.

For liquid chromatography‐tandem mass spectrometry (LC‐MS/MS) mass spectrometry, fibrinogen (digested in solution as above) was run on an 8% BIS‐TRIS gel with MES (2‐(N‐morpholino)ethanesulfonic acid) buffer (Invitrogen) under reducing conditions. Proteolytic fragments were excised from the gel and an in‐gel trypsin digest was performed as per the manufacturer's guidelines (Promega, Madison, WI, USA). Samples were analyzed on a Micromass QToF Premier with MAssLynx 4.1 software.

### Fibrin formation and fibrinolysis assays

Turbidity assays of fibrin formation and fibrinolysis were performed as previously described 25. Briefly, normal human plasma, diluted 1 : 2 in HEPES buffer containing 20 mm CaCl_2,_ was incubated in a clear 96‐well plate for 20 min at 37 °C with and without ADAMTS‐13. Fibrin formation was initiated by the addition of 2 nm human thrombin (Sigma‐Aldrich) and was followed by absorbance at 405 nm at 15‐s intervals for 60 min using a FLUOstar Omega (BMG Labtech, Ortenberg, Germany) plate reader maintained at 37°C.

For SDS PAGE of fibrin cross‐linking, 5 μm human fibrinogen was pre‐incubated with and without 50 nm ADAMTS‐13 for 1 h at 37° before the addition of 2 nm thrombin and 20 mm CaCl_2_. Cross‐linking of fibrin in these samples was allowed to proceed for either 20 min or 1 h before the sample was solubilized by the addition of 4% SDS and 2% β‐mercaptoethanol.

Fibrin formed, as above, in the absence of ADAMTS‐13 was used to determine whether ADAMTS‐13 is capable of proteolysing cross‐linked fibrin. Fibrin formation was allowed to proceed for 30 min at 37 °C following the addition of 2 nm thrombin, in clear 96‐well plates. Wells were then overlaid with either 1 μg mL^−1^ tissue‐type plasminogen activator (t‐PA) (Sigma‐Aldrich) or 50 nm ADAMTS‐13 and the absorbance at 405 nm was recorded at 60‐s intervals for 180 min.

To determine the effect of ADAMTS‐13 proteolysis of fibrinogen on fibrinolysis, fibrin was formed in the presence of 100 ng mL^−1^ t‐PA after pre‐incubation with and without ADAMTS‐13. Fibrin formation/lysis was determined by measuring the absorbance at 405 nm at 15‐s intervals for 30 min. Lysis times were calculated from the two points of 50% maximal absorbance.

### Permeation assay

Permeation assays were performed as previously described 26. Fibrin formation was initiated by the addition of 2 nm thrombin to normal human plasma and pre‐incubated with and without 50 nm ADAMTS‐13, in 10 mL disposable columns (Biorad, Hercules, CA, USA). After 30 min at 37 °C the 200‐μL fibrin bed was topped with HEPES buffer and the volume of buffer passing through the column was manually recorded at 10‐min intervals.

### Confocal microscopy

Normal human plasma, supplemented with 150 μg mL^−1^ AlexaFluor594 labelled human fibrinogen (Invitrogen), was pre‐incubated with and without 50 nm GoF ADAMTS‐13 for 40 min at 37 °C. Fibrin formation was performed in glass chamber slides (Ibidi GmbH, Planegg, Germany), by the addition of 2 nm thrombin and incubation at 37 °C for 30 min. Images were acquired using a Zeiss LSM780 (Carl Zeiss AG Oberkochen, Germany) confocal microscope with a Plan‐Apo 10x/0.45 objective and processed using FIJI imaging software (ImageJ, Madison, WI, USA).

### *In vitro* thrombosis model

Vena8 Fluoro + biochips (Cellix) were coated with 200 μg mL^−1^ collagen type III (Southern Biotech, Birmingham, AL, USA) and 100 pm tissue factor (Sigma‐Aldrich) before being blocked with coagulation buffer (1% bovine serum albumin, 75 mm CaCl_2_, 37.5 mm MgCl_2_ in HEPES buffer). Whole human blood was collected on 129 mm trisodium citrate (1 : 10 dilution). Platelets were labelled with 10 μm DiOC_6_ (Sigma‐Aldrich) and 100 μg mL^−1^ AlexaFluor594 labelled fibrinogen (Invitrogen) was added to visualize fibrin formation. Citrated blood was diluted 9 : 1 with coagulation buffer immediately before perfusion over the collagen surface at 1500 s^−1^ for 3 min. This was repeated three times to provide uninterrupted flow of coagulating blood for sufficient time to allow the formation of stable fibrin clots.

This was followed by a further 5‐min perfusion with blood, collected on D‐Phenylalanyl‐prolyl‐arginyl Chloromethyl Ketone (PPACK) (Sigma‐Aldrich) and enoxaparin (low‐molecular‐weight heparin from Sanofi‐aventis, Bridgewater, NJ, USA), and supplemented with DiOC_6_ labelled platelets, labelled fibrinogen and increasing concentrations of ADAMTS‐13. At the end of this period the fluorescence intensity of DiOC_6_ platelets and AlexaFluor594 fibrin was measured at multiple locations along the biochip channel and used to determine the EC_50_ of ADAMTS‐13 cleavage of fibrin clots.

## Results

### Conformationally active ADAMTS‐13 (*ca*ADAMTS‐13) proteolyses the Aα chain of fibrinogen

**Figure 1:**
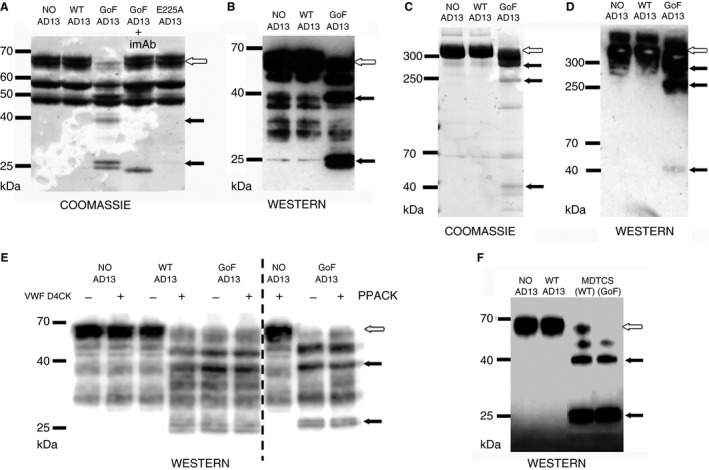
ADAMTS‐13 proteolysis of fibrinogen determined by SDS PAGE and Western blot. Purified human fibrinogen was incubated at 37 °C with 50 nm purified ADAMTS‐13 or MDTCS. Samples taken after 180 min were run on SDS PAGE under reducing (A, B, E and F) and non‐reducing conditions (C and D) and analyzed by Coomassie staining (A and C) and by Western blot using a pAb against the Aα chain of fibrinogen (B, D, E and F). Intact fibrinogen (C and D) and the Aα chain (A, B, E and F) are indicated by white arrows. Proteolytic fragments are indicated by black arrows. Fibrinogen proteolysis by wild‐type (WT) ADAMTS‐13 is only observed after a pre‐incubation with the VWF D4CK domain fragment (E). Proteolysis is completely inhibited when ADAMTS‐13 is pre‐incubated with an inhibitory mAb (imAb) targeted against the metalloprotease domain (3H9) (A). Proteolysis of fibrinogen is not inhibited by the serine protease inhibitor D‐Phenylalanyl‐prolyl‐arginyl Chloromethyl Ketone (PPACK) (E).

On SDS PAGE, under non‐reducing conditions, fibrinogen migrated as a single, broad band at approximately 340 kDa (Fig. 1C and D). When reduced, the Aα, Bβ and γ chains of fibrinogen were resolved at 65, 56 and 47 kDa, respectively (Fig. 1A and B). When fibrinogen was pre‐incubated with wild‐type (WT) ADAMTS‐13, or an inactive active site variant (E225A ADAMTS‐13), there was no observable alteration of these migration patterns. However, when pre‐incubated with the GoF ADAMTS‐13 variant (Fig. 1A–D), WT ADAMTS‐13 that had been pre‐incubated with the VWF D4CK domain fragment (Fig. 1E) or the C‐terminal truncated (*ca*) variants, WT and GoF MDTCS (Fig. 1F), proteolysis of fibrinogen was observed. This resulted in a depletion of intact 340 kDa fibrinogen, observed under non‐reducing conditions, and depletion of the intact Aα chain observed under reducing conditions. Proteolysis of the Aα chain released a 40 kDa fragment from fibrinogen, reducing its size to 290 kDa and then 250 kDa. Under reducing conditions, both this 40 kDa fragment and the remaining 25 kDa Aα chain fragment were detected. All of these proteolytic fragments were detected on Western blot using a pAb raised against the Aα chain. Proteolysis was abolished in the presence of an inhibitory mAb (imAb, 3H9), directed against the ADAMTS‐13 metalloprotease domain (Fig. 1A) but was not inhibited by the serine protease inhibitor PPACK (Fig. 1E).

**Figure 2:**
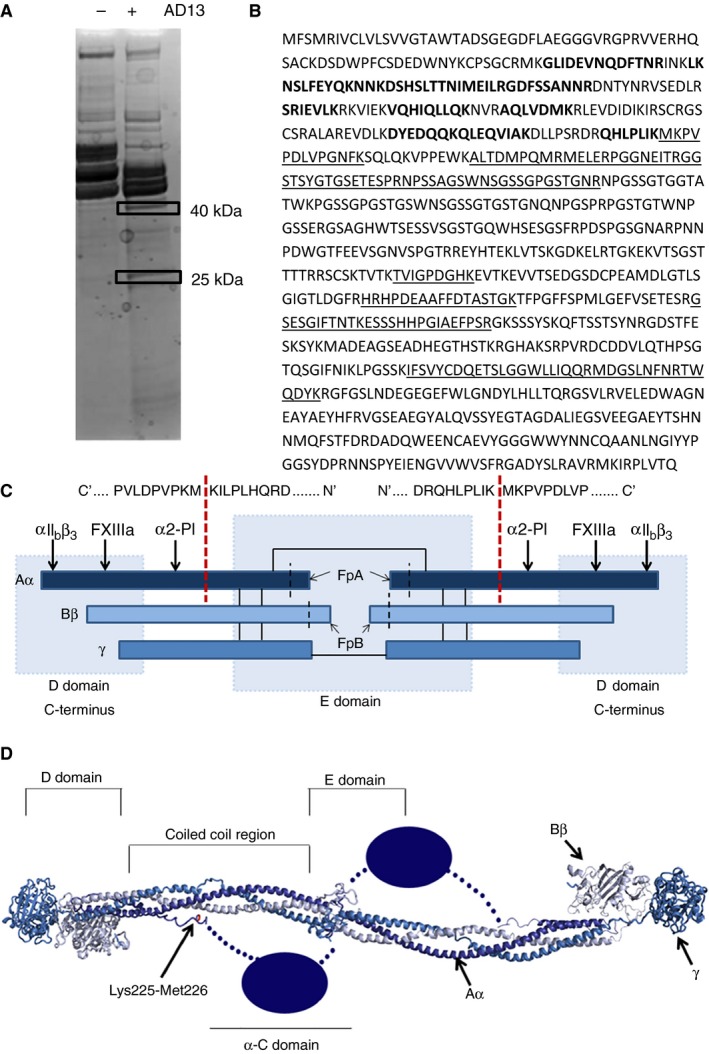
LC‐MS/MS of fibrinogen cleavage products. (A) Purified human fibrinogen was incubated overnight at 37 °C with or without 50 nm purified gain of function (GoF) ADAMTS‐13. The proteolysis reactions were separated by SDS PAGE under reducing conditions. The two proteolytic fragments of the Aα chain (40 and 25 kDa) were excised and subjected to an in‐gel trypsin digestion. (B) The resulting peptides were sequenced by liquid chromatography‐tandem mass spectrometry (LC‐MS/MS), identified against Swissprot entry P02671 (FIBA_HUMAN) and mapped onto the fibrinogen Aα sequence. Sequences in bold were identified in the 25 kDa fragment and those that are underlined were identified in the 40 kDa fragment. (C) The domain structure of human fibrinogen is determined by interchain disulphide bonds between the Aα, Bβ and γ chains (solid black lines). Thrombin cleavage occurs at the N‐terminus of the Aα and Bβ chains (black dashed lines) liberating fibrinopeptide A (FpA) and fibrinopeptide B (FpB). ADAMTS‐13 proteolysis results in a 40 kDa C‐terminal cleavage fragment of the Aα chain (bold dashed lines) as determined by SDS PAGE (Fig. 1) and MALDI TOF mass spectrometry. Using LC‐MS/MS analysis of the cleavage fragments (supplementary Tables S1 and S2) the site of proteolysis has been determined to be between Lys225 and Met226. Therefore the released cleavage fragment contains αIIbβ3, FXIIIa and α2‐antiplasmin binding sites. (D) The Lys225‐Met226 bond is situated in the protease sensitive hinge region of the Aα chain, which is only partially resolved in this crystal structure of human fibrinogen (3GHG) 28. The unresolved flexible α‐C domain, represented here in blue, is completely removed by GoF ADAMTS‐13 proteolysis.

Further analysis of the fibrinogen Aα proteolytic fragments was performed using LC‐MS/MS. Both fragments (40 kDa and 25 kDa), resulting from an in‐solution digestion with GoF ADAMTS‐13, were resolved by SDS PAGE (Fig. 2A). These bands were excised and subjected to an in‐gel digestion with trypsin. The resulting peptides were sequenced by LC‐MS/MS (Tables S1 and S2) and mapped onto the fibrinogen Aα amino acid sequence (Fig. 2B). Coverage of the fibrinogen Aα sequence in the 25 kDa fragment was limited to the N‐terminal residues up to, and including, Lys225. Peptides identified in the 40 kDa fragment were mapped exclusively to the C‐terminal portion of the sequence, beginning at Met226. This indicated that the site of ADAMTS‐13 proteolysis was the Lys225‐Met226 peptide bond (Fig. 2C), which is positioned in the protease‐sensitive hinge region of the Aα chain (Fig. 2D). The lysine residue in the P1 position is unusual, but when the ADAMTS‐13 proteolysis site in the short VWF A2 domain fragment VWF115 (Tyr1605‐Met1606) was mutated to represent the site of proteolysis in fibrinogen (Tyr1605Lys VWF115), proteolysis still occurred (Fig. S1).

### *ca*ADAMTS‐13 proteolysis of fibrinogen alters fibrin formation and clot structure

**Figure 3:**
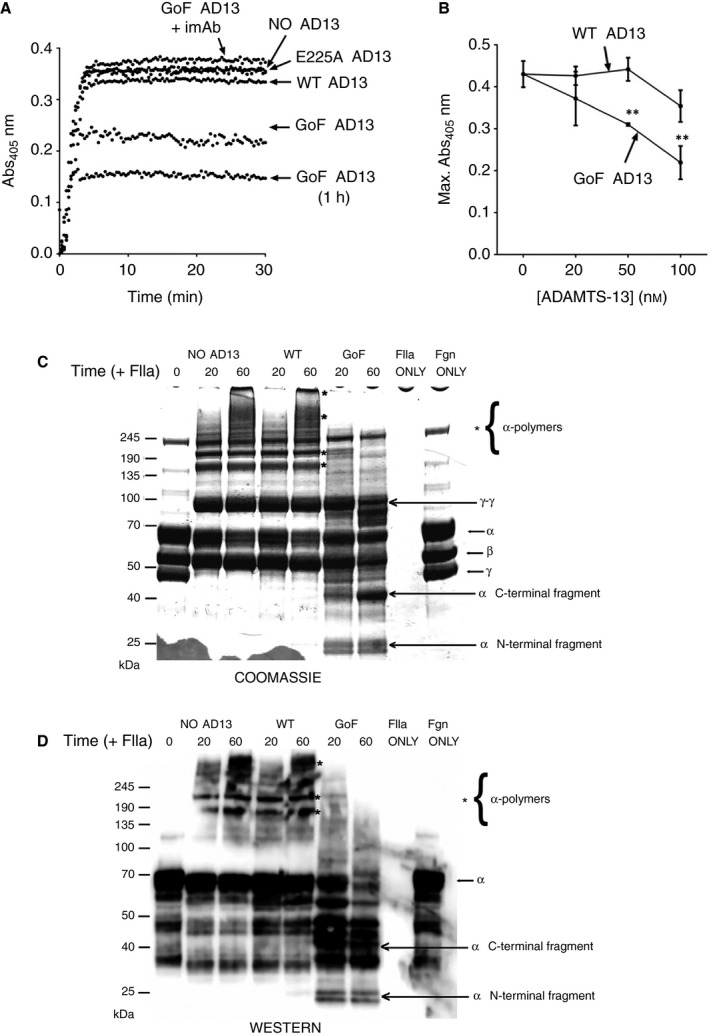
ADAMTS‐13 proteolysis of fibrinogen alters fibrin formation. (A) The formation of fibrin in normal human plasma, with and without pre‐incubation with 50 nm ADAMTS‐13, was initiated by the addition of 2 nm thrombin and determined by increased absorbance at 405 nm (representative of *n* = 5). Unless indicated, the duration of the pre‐incubation was 20 min. (B) Fibrin formation assays were performed in the presence of a range of ADAMTS‐13 concentrations and used to construct dose‐response curves (mean ± SEM, *n* = 3, **P < 0.005). (C and D) Polymerization of purified fibrinogen (with and without pre‐incubation with ADAMTS‐13) was initiated by the addition of 2 nm thrombin and allowed to proceed for either 0, 20 or 60 min before sample solubilization. To examine the extent of fibrin cross‐linking, samples were run on SDS PAGE for either Coomassie staining (C) or Western blot using a pAb against the fibrin(ogen) α chain (D).

The formation of fibrin in plasma, or in a purified component assay, following the addition of thrombin can be determined by an increase in absorbance at 405 nm 25. Neither the extent of this absorbance change or the rate at which it occurs was altered when plasma was pre‐incubated for 20 min with WT ADAMTS‐13 or the inactive active site variant E225A ADAMTS‐13 (Fig. 3A). However, pre‐incubation with GoF ADAMTS‐13 resulted in a decrease in the maximal absorbance. The extent of this decreased absorbance was dose dependent (Fig. 3B), was proportional to the duration of pre‐incubation with GoF ADAMTS‐13 (Fig. 3A) and was abolished in the presence of an inhibitory mAb against ADAMTS‐13 (Fig. 3A).

The decrease in absorbance in the presence of WT ADAMTS‐13 (Fig. 3B), even at high concentrations, was insignificant (*P* > 0.06). However, in the presence of 50 nm GoF ADAMTS‐13, there was a progressive and significant (*P* < 0.01) decrease in absorbance. This demonstrates that the proteolysis of fibrinogen in plasma, and the resulting alteration of fibrin formation, were dependent on ADAMTS‐13 conformation.

Stabilization of fibrin monomers, firstly through γ‐γ dimer formation and later through α chain cross‐linking, can be visualized by SDS PAGE 27. Upon proteolysis of fibrinogen by thrombin there was a shift in the migration of the Aα and Bβ chains corresponding to the release of the fibrinopeptides FpA and FpB (Fig. 3C). Following activation of factor (F) XIII by thrombin, both γ‐γ dimers and lower order α‐polymers were formed within 20 min (Fig. 3C). Within 1 h, higher order α‐polymers were formed. This was also the case for fibrinogen that had been pre‐incubated with WT ADAMTS‐13 before the addition of thrombin (Fig. 3C). However, proteolysis of the Aα chain of fibrinogen during pre‐incubation with GoF ADAMTS‐13, indicated by the appearance of N‐terminal and C‐terminal Aα chain fragments, resulted in reduced α‐polymer formation (Fig. 3C and D).

**Figure 4:**
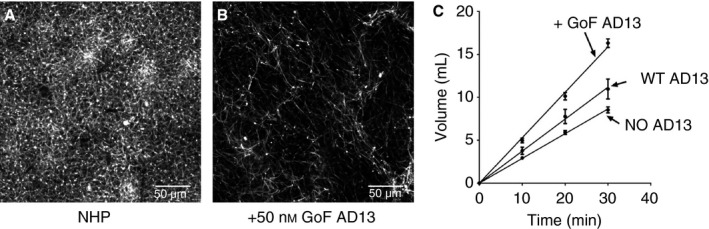
Confocal microscopy of fibrin formation in normal human plasma. (A and B) Fibrin formation was initiated by the addition of 2 nm thrombin to normal human plasma, supplemented with AlexaFluor594 fibrinogen, following a 20 min pre‐incubation with and without gain of function (GoF) ADAMTS‐13. Images are representative and scale bars represent 50 μm. (C) Clot permeation assays were performed on fibrin clots formed using normal human plasma in the absence and presence of ADAMTS‐13. Values are mean ± SEM, *n* = 3.

The reduced absorbance change in fibrin formation assays, and reduced α‐polymer formation indicated by SDS PAGE, suggested that proteolysis of fibrinogen by *ca*ADAMTS‐13 would alter the density of the fibrin network in clotting plasma. This was confirmed by confocal microscopy of the fibrin network formed in the absence (Fig. 4A) and presence (Fig. 4B) of GoF ADAMTS‐13. In ADAMTS‐13‐treated samples, fibrin appeared to be less dense, with reduced lateral association resulting in larger pores. This was also reflected in the increased permeability of these clots (Fig. 4C).

### *ca*ADAMTS‐13 proteolysis of fibrinogen increases the susceptibility of fibrin to plasmin cleavage

**Figure 5:**
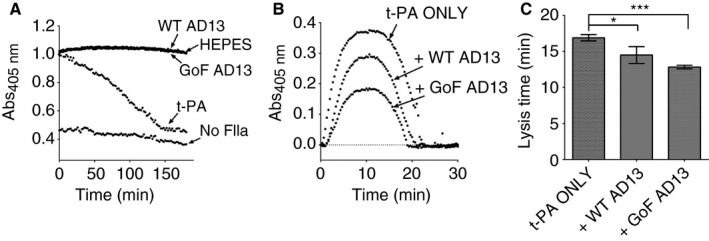
ADAMTS‐13 proteolysis of fibrinogen increases the susceptibility of fibrin to fibrinolysis. (A) ADAMTS‐13 is unable to proteolyse cross‐linked fibrin. (B) Clot lysis assays, in which fibrin formation in human plasma is initiated in the presence of 100 ng mL^−1^ tissue‐type plasminogen activator (t‐PA), were performed with and without pre‐incubation with ADAMTS‐13. (C) Clot lysis times (mean ± SEM, *n* = 7) confirm an increased susceptibility of fibrin to fibrinolysis by plasmin when fibrinogen is pre‐incubated with gain of function (GoF) ADAMTS‐13 (*P < 0.05, ***P < 0.001).

Once fully cross‐linked, 30 min after thrombin addition, fibrin appeared to be resistant to proteolysis by *ca*ADAMTS‐13 (Fig. 5A). Accordingly, fibrin formed following pre‐incubation with GoF ADAMTS‐13, despite being less dense, did not appear to be proteolysed within the course of fibrin formation assays (Fig. 3A). However, when fibrin was formed in the presence of ADAMTS‐13 and t‐PA the lysis time was reduced (Fig. 5B) to 14.5 ± 1.2 min (*P* < 0.05) and 12.8 ± 0.2 min (*P* < 0.001) for WT and GoF ADAMTS‐13, respectively, compared with 16.9 ± 0.4 min for t‐PA alone (Fig. 5C).

### *ca*ADAMTS‐13 proteolysis of fibrinogen increases the rate of clearance of fibrin thrombi *in vitro*

**Figure 6:**
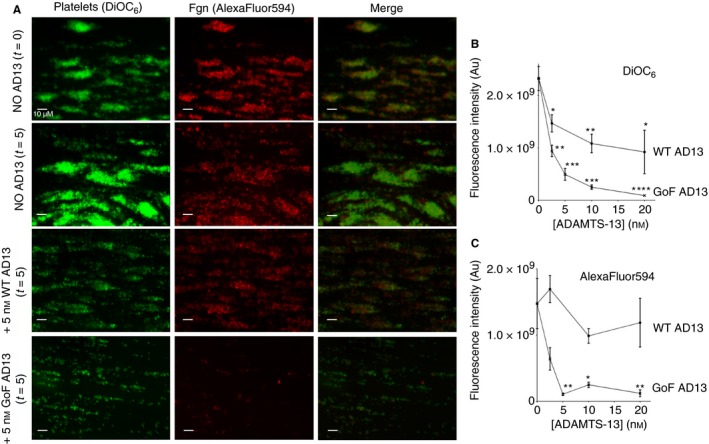
ADAMTS‐13 clearance of platelet‐rich fibrin thrombi. (A) Platelet‐rich fibrin thrombi were formed on a prothrombotic surface and the labelled platelets and fibrin(ogen) were imaged at *t* = 0 (top panel). Subsequently the thrombi were perfused with blood with and without wild‐type (WT) or gain of function (GoF) ADAMTS‐13 and image acquisition was repeated at *t* = 5 min. (B and C) Residual platelet (DiOC6) or fibrinogen (AlexaFluor594) fluorescence was recorded after 5 min perfusion at a range of ADAMTS‐13 concentrations to derive EC50 values. Images are representative and fluorescence values are mean ± SEM, *n* = 3 (*P < 0.05, **P < 0.005, ***P < 0.001, ****P < 0.0001).

The consequence of fibrinogen proteolysis by *ca*ADAMTS‐13 for the formation of a stable thrombus has been examined using an *in vitro* perfusion model. In the absence of ADAMTS‐13, the large fibrin and platelet‐rich thrombi, formed during an initial 9‐min perfusion period, remained stable throughout the second perfusion period (Fig. 6A). When ADAMTS‐13 was present during the second period of perfusion, there was a dose‐dependent reduction in the residual platelet fluorescence (Fig. 6A and B). The EC_50_ values of WT ADAMTS‐13 and GoF ADAMTS‐13 in this assay, in terms of platelet clearance, were 11.2 ± 2.8 nm and 1.5 ± 0.9 nm, respectively.

WT ADAMTS‐13 had little effect on the fibrin component of these thrombi; the reduction in fibrin fluorescence was not significant (*P* > 0.2) even at concentrations above 10 nm (Fig. 6C). GoF ADAMTS‐13, on the other hand, induced significant reduction of fibrin fluorescence (*P* < 0.05) at all concentrations above 2.5 nm. The EC_50_ values of WT ADAMTS‐13 and GoF ADAMTS‐13 in this assay, in terms of fibrin clearance, were 25.2 ± 9.7 nm and 3.0 ± 1.7 nm, respectively.

## Discussion

### ADAMTS‐13 proteolysis of fibrinogen is conformation dependent

ADAMTS‐13 is capable of proteolysing the Aα chain of human fibrinogen between Lys225 and Met226, a site suggested by the partial crystal structure of human fibrinogen 28 to be surface exposed and accessible (Fig. 2D). This cleavage site bears some resemblance to the scissile bond at Tyr1605‐Met1606 of VWF, which is proteolysed by ADAMTS‐13. Both have methionine in the P1’ position, leucine in the P3 position and small, hydrophobic residues in the P2 position (valine in VWF and isoleucine in fibrinogen). When Tyr1605 in a short VWF substrate (VWF115) is mutated to lysine, proteolysis by ADAMTS‐13 still occurs (Fig. S1). This concurs with previous studies of substrate specificity of ADAMTS‐13, in which the substitution of Tyr1605 to lysine did not abolish the proteolysis of a VWF73 expressing phage 29, 30. Our findings suggest proteolysis of fibrinogen by GoF ADAMTS‐13 will be less efficient than its cleavage of unfolded VWF. Proteolysis of Tyr1605Lys VWF115 by GoF ADAMTS‐13 is incomplete within 120 min, compared with the complete proteolysis within 60 min observed in WT VWF115 cleavage assays (Fig. S1).

Importantly, we have identified proteolytic activity against fibrinogen using the GoF ADAMTS‐13 variant (Fig. 1A–D), in which the autoinhibitory spacer‐CUB domain interaction is abolished, and the truncated MDTCS variants (Fig. 1F), which lack the C‐terminal domains 22. Therefore, it appears that only ADAMTS‐13 variants that are in a pre‐activated conformation are capable of fibrinogen proteolysis. WT ADAMTS‐13, even at high concentrations (200 nm), does not exhibit proteolytic activity against fibrinogen (Fig. S5). However, upon activation by the VWF D4CK domain fragment, WT ADAMTS‐13 attains proteolytic activity against fibrinogen (Fig. 1E). This strongly suggests that WT ADAMTS‐13 in its closed conformation would not proteolyse fibrinogen in circulation, despite the high concentration of this potential substrate. Under flow, in *in vitro* models of thrombus formation, WT ADAMTS‐13 does not appear to significantly affect the stability of deposited fibrin (Fig. 6). Conformational activation of ADAMTS‐13 therefore serves as a mechanism to protect fibrinogen (and possibly other plasma proteins) from off‐target proteolysis by ADAMTS‐13.

It is possible that once conformationally activated, by VWF recruited at sites of vascular injury, ADAMTS‐13 may potentially engage and proteolyse fibrinogen. However, this may be unlikely. In the experiments described in Figs 1, 2, 3, 4, 5, ADAMTS‐13 was used at 50 nm (unless otherwise stated) and is therefore appreciably higher than the normal plasma concentration of ADAMTS‐13, 4.1 to 7.9 nm 31. We suggest that proteolysis of fibrinogen by ADAMTS‐13 may only be physiologically relevant, in the context of a possible high local concentration of ADAMTS‐13 at sites of vascular injury, where VWF induces conformation activation.

### *ca*ADAMTS‐13 proteolysis inhibits cross‐linking of fibrin and alters clot structure

The functional consequence of fibrinogen proteolysis by GoF ADAMTS‐13 is clear when examining fibrin formation. Proteolysis of the N‐terminus of fibrinogen by thrombin appears to be unaffected by proteolysis of the Aα chain C‐terminus by GoF ADAMTS‐13 (Fig. 3C). Fibrin formed from fibrinogen following *ca*ADAMTS‐13 proteolysis, appears to form normal γ‐γ dimers (Fig. 3C), although there remains some residual non‐cross‐linked γ chain indicating that the rate of γ‐γ dimer formation may be marginally reduced compared with control fibrin. This may be the result of the removal of the FXIIIa binding site from the fibrinogen Aα chain residues 371–425 32 by *ca*ADAMTS‐13 proteolysis (Fig. 2C), as this interaction has been shown to play an important regulatory role in the FXIIIa B‐subunit dissociation 33.

More importantly, *ca*ADAMTS‐13 proteolysis of fibrinogen directly reduces cross‐linking of the α chains of fibrin (Fig. 3C and D). Following a 60‐min pre‐incubation of fibrinogen with GoF ADAMTS‐13, minimal low‐order α‐polymers are formed within 20 min of thrombin addition, compared with control samples. This residual polymer formation probably arises from the small proportion of fibrinogen left intact following the incubation with ADAMTS‐13. Proteolysis of fibrinogen by GoF ADAMTS‐13 removes the α‐chain residues 241–476 and 518–584 (Fig. 2C), which are required for α‐chain association 34.

As a result of reduced α chain cross‐linking, fibrin formation is altered in plasma treated with GoF ADAMTS‐13 (Fig. 3A and Fig. 4B). The fibrin formed in these assays still polymerizes, through γ‐γ dimerization; however, the lack of α‐polymer formation results in decreased maximal absorbance, which is indicative of a lower density fibrin network. Visualization of the fibrin network by confocal microscopy confirms a pronounced decrease in the density of the fibrin network in GoF ADAMTS‐13‐treated plasma (Fig. 4B). Fibres in these samples appear to be longer and less densely packed compared with normal plasma (Fig. 4A). Even under visual inspection, clots formed in GoF ADAMTS‐13‐treated plasma appear less opaque and less viscous. There is an apparent increase in pore size, which may account for the significant increase in the permeability of these clots (Fig. 4C).

### *ca*ADAMTS‐13 proteolysis of fibrinogen increases t‐PA‐induced lysis of fibrin

The altered formation of fibrin that results from GoF ADAMTS‐13 proteolysis of fibrinogen (Fig. 3) appears to render fibrin more susceptible to the action of t‐PA/plasminogen (Fig. 5), significantly reducing the lysis time by almost 25%. By removing the potential for α‐chain cross‐linking, and by decreasing the density of the fibrin network, ADAMTS‐13 proteolysis may allow easier access for plasmin to the coiled coil regions 35. This may be compounded by the loss of the α2‐antiplasmin cross‐linking site at Lys303 36 of the fibrinogen Aα chain, following proteolysis (Fig. 2C).

Unexpectedly, given that WT ADAMTS‐13 exhibits no detectable proteolytic activity against fibrinogen, fibrin also appears to be more susceptible to plasmin in samples treated with WT ADAMTS‐13 (Fig. 5). There is evidence to suggest that WT ADAMTS‐13 is able to bind to fibrinogen with moderate affinity, which is enhanced in conformationally active variants (Fig. S3). Therefore, even in the absence of proteolytic activity, binding of ADAMTS‐13 may hinder plasmin cleavage of fibrin.

### *ca*ADAMTS‐13 proteolysis of fibrinogen enhances platelet and fibrin clearance in *in vitro* models of thrombosis

In *in vitro* models of thrombosis WT ADAMTS‐13 is known to reduce VWF‐mediated platelet recruitment to a prothrombotic surface 37. It also prevents further recruitment of platelets to preformed fibrin/platelet‐rich thrombi (Fig. 6B). In this assay the EC_50_ of the GoF ADAMTS‐13 variant, in terms of platelet clearance, is more than seven times lower than that of WT ADAMTS‐13. The efficacy of GoF ADAMTS‐13 in this assay is higher than expected, given that the variant exhibits only a 2.5‐fold increase in proteolytic activity against VWF in static assays 22 and only a ~3‐fold decease in EC_50_ in platelet recruitment assays (Fig. S2). The maximal effect of WT ADAMTS‐13, which acts only on VWF‐mediated platelet capture, in this assay is a ~50% decrease in platelet coverage. This suggests the remaining 50% of platelets are bound to fibrinogen. The increased clearance of platelets by GoF ADAMTS‐13 may be the result of fibrinogen proteolysis, which removes a αII_b_β_3_ binding site on the fibrinogen Aα chain (Fig. 2C), thereby reducing fibrinogen‐mediated platelet–platelet interactions in the developing thrombus 38. This is supported by the fact that the GPIIbIIIa receptor antagonist GRR144053 induces a similar decrease in platelet coverage (~60%) when perfused over the preformed thrombi in this assay (Fig. S4).

Moreover, GoF ADAMTS‐13 is capable of reducing the fibrin component of these preformed thrombi (Fig. 6C), presumably because of the decreased density and increased permeability of the fibrin network formed in GoF ADAMTS‐13‐treated plasma. WT ADAMTS‐13 does not seem to exhibit any significant effect on the fibrin component of the thrombi in these assays (Fig. 6C), further supporting the hypothesis that the proteolytic activity of ADAMTS‐13 against fibrinogen is dependent on conformation.

### Off‐target proteolysis may be an important consideration in the development of ADAMTS‐13‐based therapies

The therapeutic potential of recombinant WT ADAMTS‐13 has already been examined in murine models of thrombosis 39, 40, ischemic stroke 41 and myocardial ischemia 42. That GoF ADAMTS‐13 exhibits proteolytic activity against fibrinogen, as well as enhanced activity against VWF, because of its pre‐activated conformation, could alter its therapeutic potential. Many of these *in vivo* models involve the use of FeCl_3_ or occlusive filaments. In FeCl_3_‐based models the thrombus development following injury is largely VWF dependent and the thrombi formed are poor in fibrin 43. In the transient ischemia models of stroke and myocardial ischemia, infarction is largely dependent on the inflammatory response to reperfusion injury 42. Therefore, these models are unlikely to illustrate the full potential of GoF ADAMTS‐13 in thrombus resolution. In experimental 44, 45, 46 and human situations of pathological clot formation we anticipate that GoF ADAMTS‐13 will have increased efficacy compared with WT ADAMTS‐13 because of its dual antiplatelet and fibrinolytic functions.

As well as the treatment of stroke 41 and myocardial infarction 42, the use of recombinant ADAMTS‐13 as a replacement therapy in acquired TTP 47 and the possibility of gene therapies to overcome ADAMTS‐13 deficiency 48 are also being investigated. Conformationally active ADAMTS‐13 variants, such as the truncated variant MDTCS used in one gene therapy investigation 48, although having the potential for improved efficacy, may also carry the risk of off‐target proteolysis, particularly when administered at *supra*‐physiological concentrations.

## Addendum

K. South and M. O. Freitas performed the research. K. South and D. A. Lane designed the research, analyzed data, interpreted data, generated figures and wrote the paper. All authors read and approved the final version of the manuscript.

## Disclosure of Conflict of Interests

The authors state that they have no conflict of interest.

## Supporting information


**Fig. S1.** Proteolysis of Tyr1605Lys VWF115 by ADAMTS‐13.
**Fig. S2**. von Willebrand factor (VWF)‐ mediated platelet deposition under flow.
**Fig. S3.** The binding of ADAMTS‐13 to human fibrinogen.
**Fig. S4.** Displacement of αII_b_β_3_ bound platelets in preformed platelet‐rich fibrin thrombi by the Arginylglycylaspartic acid peptide GR144053.
**Fig. S5.** Proteolytic activity of wild‐type (WT) ADAMTS‐13 against fibrinogen cannot be induced by increased enzyme concentration.
**Table S1.** liquid chromatography‐tandem mass spectrometry (LC‐MS/MS) of fibrinogen 40 kDa cleavage product.
**Table S2.** liquid chromatography‐tandem mass spectrometry (LC‐MS/MS) of fibrinogen 25 kDa cleavage product.
