# Supplementary material for: Conformational quiescence of ADAMTS‐13 prevents proteolytic promiscuity
Source: J Thromb Haemost. 2016 Sep 23;14(10):2011–22. doi: 10.1111/jth.13445 (PMC5111603; doi:10.1111/jth.13445)
Supplement: Supplementary file 1 — Fig. S1. Proteolysis of Tyr1605Lys VWF115 by ADAMTS‐13. Fig. S2. von Willebrand factor (VWF)‐ mediated platelet deposition under flow. Fig. S3. The binding of ADAMTS‐13 to human fibrinogen. Fig. S4. Displacement of αIIbβ3 bound platelets in preformed platelet‐rich fibrin thrombi by the Arginylglycylaspartic acid peptide GR144053. Fig. S5. Proteolytic activity of wild‐type (WT) ADAMTS‐13 against fibrinogen cannot be induced by increased enzyme concentration. Table S1. liquid chromatography‐tandem mass spectrometry (LC‐MS/MS) of fibrinogen 40 kDa cleavage product. Table S2. liquid chromatography‐tandem mass spectrometry (LC‐MS/MS) of fibrinogen 25 kDa cleavage product. [file JTH-14-2011-s001.docx]

**Supporting information**





**Figure S1. Proteolysis of Tyr1605Lys VWF115 by ADAMTS13**. The VWF A2 domain fragment VWF115 (VWF residues 1554-1668), and its variant Tyr1605Lys, were expressed and purified as previously described [20]. Cleavage of VWF115 by ADAMTS13 was carried out as described [11]. VWF115 (WT or Tyr1605Lys variant) was incubated at 37°C with ADAMTS13 (WT, GoF or inactive variant E225A). Samples were taken at 60 and 120 minutes and visualised by SDS PAGE and silver staining. VWF115 (white arrow) is readily cleaved to produce 2 proteolytic fragments (black arrows). Proteolysis still occurs when the P1 residue is mutated from Tyr to Lys, as identified in the site of ADAMTS13 proteolysis of fibrinogen (Lys225-Met226).


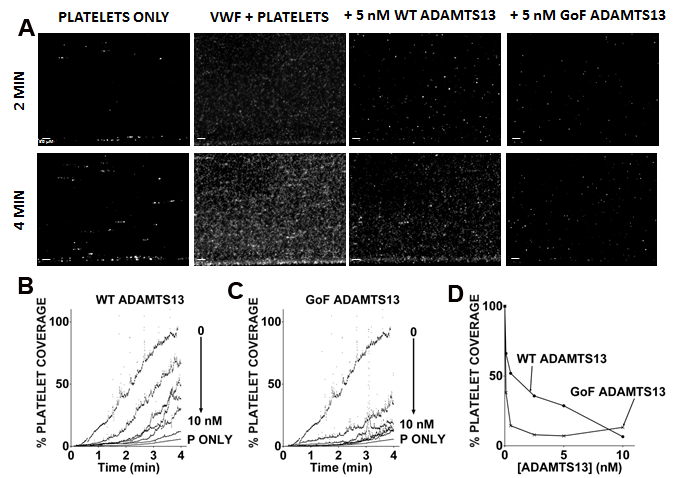


**Figure S2**. **VWF mediated platelet deposition under flow. A,** Vena8 Fluoro+ biochips (Cellix) were coated with 200 µg/ml collagen type III and blocked with 1% BSA, 1 mg/ml glucose in HEPES buffer. Washed platelets were treated with 100 nM PGE1 and 75 mU/ml Apyrase, to prevent platelet activation, before being labelled with 10 µM DiOC6. Platelets were supplemented with 10 µg/ml recombinant VWF and perfused over the collagen surface at a constant shear rate of 1500 s-1 (at which platelet capture is VWF dependant) for 4 minutes. Adhesion of labelled platelets was visualised by fluorescence imaging at 250 ms intervals using a 20x objective and analysed using Slidebook software to determine platelet coverage (%) over time. To determine the effect of ADAMTS13 on platelet capture the assay was also performed in the presence of WT or GoF ADAMTS13 (**B** and **C** respectively). **D**, EC_50_ values were determined by dose-response curves to be 0.48 ± 0.06 nM and 0.13 ± 0.06 nM for WT and GoF ADAMTS13, respectively (Values are mean ± SEM, n=3).

**Table S1. liquid chromatography-tandem mass spectrometry (LC-MS/MS) of fibrinogen 40 kDa cleavage product.**

**Table S2. liquid chromatography-tandem mass spectrometry (LC-MS/MS) of fibrinogen 25 kDa cleavage product.**

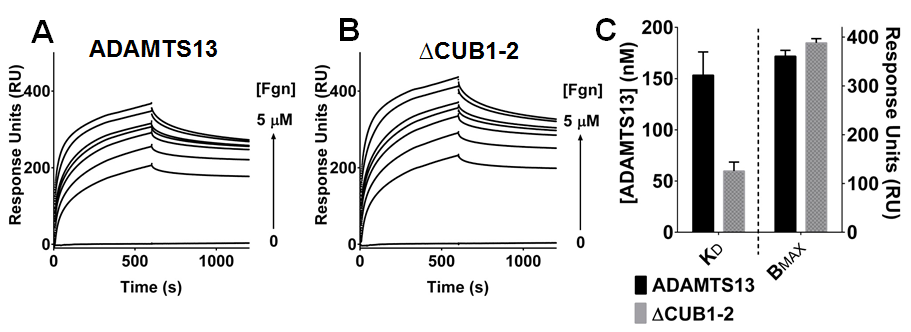


**Figure S3. The binding of ADAMTS13 to human fibrinogen.** Binding of ADAMTS13, and its truncated variant ∆CUB1-2, to human fibrinogen (Sigma) was determined by surface plasmon resonance using a Biacore T100 system (Biacore) as previously described [17]. Human fibrinogen (purified from plasma) was immobilised on an SPR chip and used to examine the binding of full length WT ADAMTS13 (**A**) or the conformationally activated truncation variant ∆CUB1-2 [22], lacking both the distal CUB domains (**B**).Values of K_D_ and B_MAX_ were determined by steady state analysis (**C**) and the results are representative of three independent experiments.

**
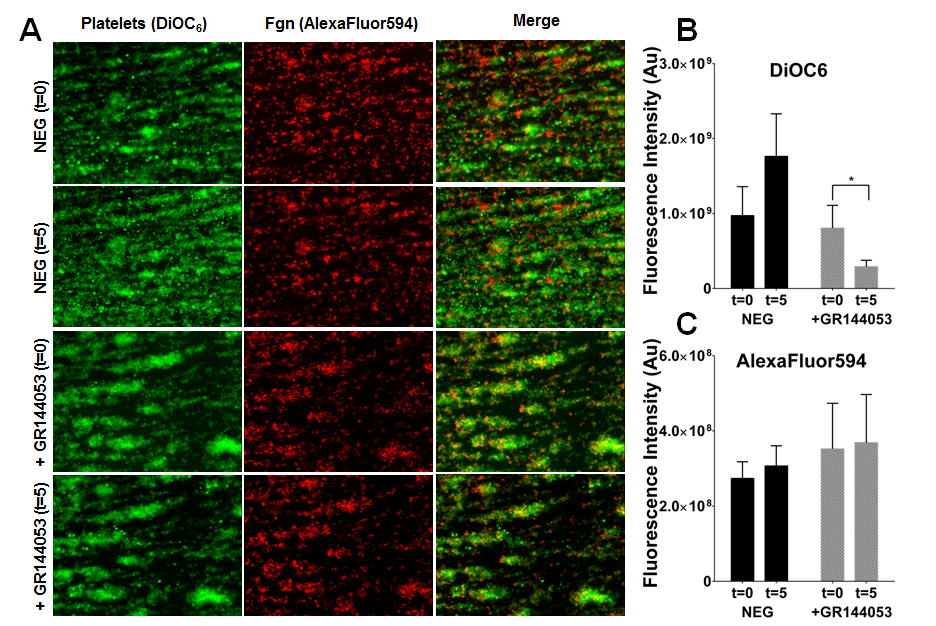
**

**Figure S4. Displacement of αII_b_β_3_ bound platelets in pre formed platelet rich fibrin thrombi by the Arginylglycylaspartic acid peptide GR144053. A,** Thrombi were formed as in the *in vitro* thrombosis model. The pre formed thrombi (t=0) were perfused with blood with/without 2 µM GR144053 for 5 minutes (t=5). **B**, in the absence of GR144053 platelets continue to be deposited but in the presence of the peptide pre-deposited platelets are displaced. **C**, the fibrin(ogen) component of the thrombi was not affected by GR144053. Images are representative and fluorescence values are mean ± SEM, n=3. (* p=0.03).

**
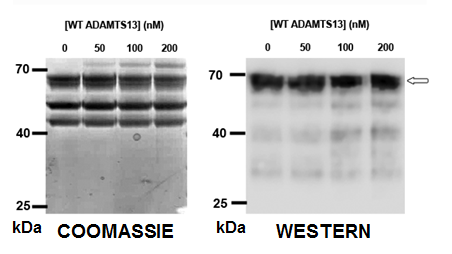
**

**Figure S5. Proteolytic activity of WT ADAMTS13 against fibrinogen cannot be induced by increased enzyme concentration.** Purified human fibrinogen was incubated at 37°C with increasing concentrations of WT ADAMTS13. Samples taken after 180 minutes were run on SDS PAGE under reducing conditions and analysed by coomassie staining and by western blot using a pAb against the Aα chain of fibrinogen**.** The intact Aα chain of fibrinogen is indicated by a white arrow.
